# Targeting TRIM54/Axin1/β-Catenin Axis Prohibits Proliferation and Metastasis in Hepatocellular Carcinoma

**DOI:** 10.3389/fonc.2021.759842

**Published:** 2021-12-09

**Authors:** Jinrong Zhu, Yongqi Wu, Shaoxi Lao, Jianfei Shen, Yijian Yu, Chunqiang Fang, Na Zhang, Yan Li, Rongxin Zhang

**Affiliations:** ^1^ Guangdong Province Key Laboratory for Biotechnology Drug Candidates, School of Life Sciences and Biopharmaceutics, Guangdong Pharmaceutical University, Guangzhou, China; ^2^ Department of Cardiothoracic Surgery, Taizhou Hospital of Zhejiang Province Affiliated to Wenzhou Medical University, Linhai, China; ^3^ Department of General Practice, Heyuan People’s Hospital, Heyuan, Guangdong, China

**Keywords:** TRIM54, ubiquitination, Wnt/β-catenin signaling, proliferation, metastasis, hepatocellular carcinoma

## Abstract

Accumulating evidence demonstrates that dysregulation of ubiquitin-mediated degradation of oncogene or suppressors plays an important role in several diseases. However, the function and molecular mechanisms of ubiquitin ligases underlying hepatocellular carcinoma (HCC) remain elusive. In the current study, we show that overexpression of TRIM54 was associated with HCC progression. TRIM54 overexpression facilitates proliferation and lung metastasis; however, inhibition of TRIM54 significantly suppressed HCC progression both *in vitro* and *in vivo*. Mechanically, we demonstrated that TRIM54 directly interacts with Axis inhibition proteins 1 (Axin1) and induces E3 ligase-dependent proteasomal turnover of Axin1 and substantially induces sustained activation of wnt/β-catenin in HCC cell lines. Furthermore, we showed that inhibition of the wnt/β-catenin signaling pathway *via* small molecule inhibitors significantly suppressed TRIM54-induced proliferation. Our data suggest that TRIM54 might function as an oncogenic gene and targeting the TRIM54/Axin1/β-catenin axis signaling may be a promising prognostic factor and a valuable therapeutic target for HCC.

## Introduction

Hepatocellular carcinoma (HCC) is now the second leading cause of cancer death worldwide and accounts for the majority of primary liver cancers (1). Despite advances in medical and surgical therapies, the outcome for HCC patients was substantially poor, with 5-year survival rates of 10%-20% (2, 3). Up to now, the late diagnosis, organ metastasis, and high rate of recurrence still mainly contributed for the dim survival for HCC patients (4). Therefore, further clarification and identification of the novel molecular markers underlying the HCC progression are urgently needed to provide a potential target for the treatment of HCC.

Ubiquitination is one of the important posttranslational modifications which play crucial roles as a regulator of target protein degradation by the proteasome system (5). Briefly, ubiquitination modification begins with the activation of Ub by the Ub-activating (E1) enzyme and is then transferred onto an E2-conjugating enzyme (6). At the same time, E3 Ub ligases interact with the Ub–E2 complex and the substrate protein and mediate an isopeptide bond formation between the C terminus of Ub and a substrate lysine (7, 8). There are more than 600 Ub E3 ligases were annotated and various types of E3 Ub ligases, such as RING E3 ligases, HECT E3 ligases, and RBR E3 ligases, which with different structures and different functions are involved in tumor development and progression (9, 10).

RING E3 ubiquitin ligases are the largest family of Ub ligase, and one of the subfamilies of RING E3 ligases is tripartite motif (TRIM)-containing proteins. There are approximately 70 known TRIM proteins in humans which are characterized by one or two zinc-binding motifs, named B-boxes, and an associated coiled-coil region (11). Most of the TRIM proteins function as E3 ubiquitin ligases, and several TRIM family members are involved in various regulated biological processes by posttranslational modifications of the ubiquitin–proteasome system (12).

It has been reported that the alteration of TRIM family proteins is involved in results in metastasis, chemotherapy resistance, and recurrence in many cancers, including HCC. For example, an elevated expression of TRIM11 correlates with poor prognosis in HCC patients and exerts its oncogenic effect in HCC by downregulating p53 both *in vitro* and *in vivo* (13, 14). Pengbo Guo and colleges reported that TRIM31 exerted its oncogenic effect and promotes anoikis resistance through promoting the E3 ligase-mediated K48-linked ubiquitination of the TSC1–TSC2 complex or regulation of the p53-AMPK axis, respectively (15, 16). Yi Wang and colleagues suggest that TRIM26 silencing can promote cancer cell proliferation, colony formation, migration, and invasion *in vitro* and determine the progression of hepatocellular carcinoma (17, 18). Moreover, other TRIM proteins, including TRIM52, TRIM25 (also known as EFP), and TRIM50, have been implicated in the progression of HCC by regulating the p53 signaling pathway and ubiquitylating the PPM1A and Keap1-Nrf2 pathway, respectively (19–21). The above studies suggest the important role of TRIM proteins in HCC development, and the identification and targeting of E3 ligases that are involved in the regulation of oncoproteins or tumor-suppressor proteins are a current focus of cancer research.

## Materials and Methods

### Cell Lines and Cell Culture

Human HCC cell lines (SK-HEP-1, Hep3B, PLC/PRF/5) were purchased from the Cell Bank of Chinese Academy of Sciences (Shanghai, China), and HCCLM3, MHCC97L, MHCC97H, and SUN182 were purchased from the American Type Culture Collection (ATCC); cells were grown in Dulbecco’s modified Eagle’s medium (DMEM, Invitrogen, Carlsbad, CA, USA) supplemented with 10% fetal bovine serum (FBS, Invitrogen), at 37°C in a 5% CO_2_ atmosphere in a humidified incubator. The human hepatic stellate cell line (LX2) was purchased from Shanghai Anwei Biotechnology Co., Ltd. (Shanghai, China), and cultured in the specific medium according to the manufacturer’s instructions. SV40 large T antigen-immortalized normal human liver epithelial cells (THLE2) (American Type Culture Collection, Manassas, VA) were cultured in LHC-8 medium supplemented with 70 ng/ml phosphoethanolamine, 5 ng/ml epidermal growth factor, 10% fetal bovine serum, and antibiotics at 37°C in a humidified atmosphere containing 95% air and 5% CO_2_. All cell lines were authenticated by short tandem repeat (STR) fingerprinting.

### Patient Information and Tissue Specimens

A total of 105 paraffin-embedded and archived HCC samples (June 2010 to June 2012) were confirmed pathologically and were collected from the tissue bank of Taizhou Hospital of Zhejiang Province. For all cases, complete follow-up data were available. Clinical information on the samples is summarized in **Supplemental Table 1**. Freshly collected HCC tissues were frozen and stored in liquid nitrogen until further use. Prior patient consent and approval from the Institutional Research Ethics Committee were obtained for the use of these clinical materials for research purposes.

### Plasmids, Virus Constructs, and Retroviral Infection of Target Cells

Human TRIM54-coding sequences were amplified by PCR and cloned into the pCDH-CMV-MCS-3Flag-EF1-CopGFP-T2A-Puro vector by multiple cloning sites (XbaI/BamHI). Human TRIM54-targeting short hairpin RNA (shRNA) oligonucleotide sequences were cloned into PLKO-U6-EGFP-P2A-PURO to generate TRIM54-shRNA. Transfection of TRIM54 plasmids or shRNA was performed using the Lipofectamine 3000 Reagent (Invitrogen, Carlsbad, CA) according to the manufacturer’s instruction. Stable cell lines expressing indicated genes were selected for 10 days with 0.5 μg/ml puromycin after infection. All the primers use in this study are indicated in **Supplemental Table 3**.

### Western Blot Analysis

Western blotting was performed according to the manufacturer’s instruction, using the primary antibodies, anti-flag (Sigma, F3165), anti-TRIM54 (Proteintech, 21074-1-AP), anti-myc (Proteintech, 16286-1-AP), anti-Axin1 (Proteintech, 16541-1-AP), anti-HA (Abcam, ab9110), and anti-β-catenin (Abcam, ab32572). Following the initial Western blot assay, the membranes were stripped and re-probed with anti-α-tubulin or anti-p84 (Sigma, Saint Louis, MO, USA) as a protein loading control.

### Immunohistochemistry

Immunohistochemical analysis was performed to detect the protein expression in 105 human HCC tissues according to the manufacturer’s instruction. Paraffin-embedded tissues were analyzed using immunohistochemistry (IHC) with anti-TRIM54, anti-β-catenin anti-PCNA, or anti-Axin1 antibody. The degree of immunostaining of formalin-fixed, paraffin-embedded sections was reviewed and scored separately by two independent pathologists uninformed of the histopathological features and patient data of the samples. The scores were determined by combining the proportion of positively stained tumor cells and the intensity of staining. The scores given by the two independent pathologists were combined into a mean score for further comparative evaluation. Tumor cell proportions were scored as follows: 0, no positive tumor cells; 1, <10% positive tumor cells; 2, 10%–35% positive tumor cells; 3, 35%–75% positive tumor cells; 4, >75% positive tumor cells. Staining intensity was graded according to the following standard: 1, no staining; 2, weak staining (light yellow); 3, moderate staining (yellow brown); 4, strong staining (brown). The staining index (SI) was calculated as the product of the staining intensity score and the proportion of positive tumor cells. Using this method of assessment, we evaluated protein expression in malignant lesions by determining the SI, with possible scores of 0, 2, 3, 4, 6, 8, 9, 12, and 16. Samples with a SI ≥ 8 were determined as high expression, and samples with a SI < 8 were determined as low expression. Cutoff values were determined on the basis of a measure of heterogeneity using the log-rank test with respect to overall survival.

### Xenografted Tumor Model, IHC, and H&E Staining

BALB/c-nu mice (4-5 weeks of age) were purchased from the Center of Experimental Animal of Guangzhou University of Chinese Medicine. For the first model, mice were randomly divided into four groups (*n* = 5/group). Each group of mice was inoculated subcutaneously with Hep3B/Vector cells (1×10^6^), Hep3B/TRIM54 cells (1×10^6^), Hep3B/control cells (1×10^6^), and Hep3B/shTRIM54 cells (1×10^6^), in the right dorsal flank per mouse. Tumors were examined twice weekly; length and width measurements were obtained with calipers, and tumor volumes were calculated using the equation (L*W^2^)/2. Serial 5.0-μm sections were cut and subjected to IHC staining using anti-PCNA antibodies. The images were captured using the DM500 Leica image analysis system. In the tail vein injection model, the indicated cells (1 × 10^6^) were injected into the tail vein of nude mice. For HCC cells expressing luciferase, bioluminescent imaging was performed using Xenogen IVIS Spectrum (Caliper Life Sciences). All experimental procedures were approved by the Institutional Animal Care and Use Committee of Guangdong Pharmaceutical University.

### Colony Formation Assay

Cells plated onto six-well plates at 0.5 × 10^3^) cells per well were cultured for 10 days. Colonies were then fixed with 10% formaldehyde for 10 min and stained for 10 min with 1.0% crystal violet. All experiments were performed in triplicates.

### Anchorage-Independent Growth Ability Assay

Cells were trypsinized, and 2 × 10^3^ cells were resuspended in 2 ml complete medium plus 0.33% agar (Sigma). The agar–cell mixture was plated on top of a bottom layer consisting of 0.66% agar in complete medium. After 10 days, colony size was measured using an ocular micrometer, and colonies larger than 0.1 mm in diameter were counted. The experiment was performed three times for each cell line.

### Invasion Assay

Cells (2 × 10^4^) were plated on the top side of a polycarbonate Transwell filter (pre-coated with Matrigel) in the upper chamber of a BioCoat Invasion Chamber (BD Biosciences) and incubated at 37°C for 22 h. The cells remaining on the upper surface were removed with cotton swabs. Cells that had migrated to the lower membrane surface were fixed in 1% paraformaldehyde, stained with hematoxylin, and counted under an optical microscope (×100 magnification). Cell counts are expressed as the mean number of cells from 10 random fields per well.

### Luciferase Assay

Three thousand cells were seeded in triplicate in 48-well plates and allowed to settle for 24 h. One hundred nanograms of luciferase reporter plasmid or the control-luciferase plasmid, plus 1 ng of pRL-TK Renilla plasmid (Promega), was transfected into cells using the Lipofectamine 2000 reagent (Invitrogen) according to the manufacturer’s recommendation. Luciferase and Renilla signals were measured 24 h after transfection using the Dual Luciferase Reporter Assay Kit (Promega) according to a protocol provided by the manufacturer.

### Immunoprecipitation and Mass Spectrometry Analysis

Immunoprecipitation and mass spectrometry analysis were performed in accordance with standard procedures. In brief, lysates from 3 × 10^7^ cells transfected with the indicated constructs were incubated with myc- or Flag-conjugated agarose beads (Sigma-Aldrich, Germany) overnight at 4°C. Beads containing affinity-bound proteins were washed six times and then eluted with 200 μl of 1 M glycine (pH 3.0) twice, and the eluates were concentrated to a volume of 30 μl. The collected proteins were separated on SDS-polyacrylamide gels, stained with Coomassie blue, and specific bands were excised and subjected to LC-mass spectrometry (MS)/MS analysis.

### Statistical Analysis

Statistical tests for data analysis included Fisher’s exact test, log-rank test, chi-square test, and Student’s two-tailed t test. Multivariate statistical analysis was performed using a Cox regression model. Statistical analyses were performed using the SPSS 21.0 statistical software package. Data represent mean ± SD. *p* < 0.05 was considered statistically significant.

### Microarray Data Process and Visualization

Microarray data were downloaded from the TCGA database (http://www.tcga.org/) and from the GEO database (https://www.ebi.ac.uk/arrayexpress/).

For analysis of the Venn diagram (http://bioinformatics.psb.ugent.be/webtools/Venn/),

GSEA was performed using GSEA 2.0.9 (http://www.broadinstitute.org/gsea/).

## Results

### TRIM54 Overexpression Correlates With Progression and Poor Prognosis in Hepatocellular Carcinoma

**Figure 1:**
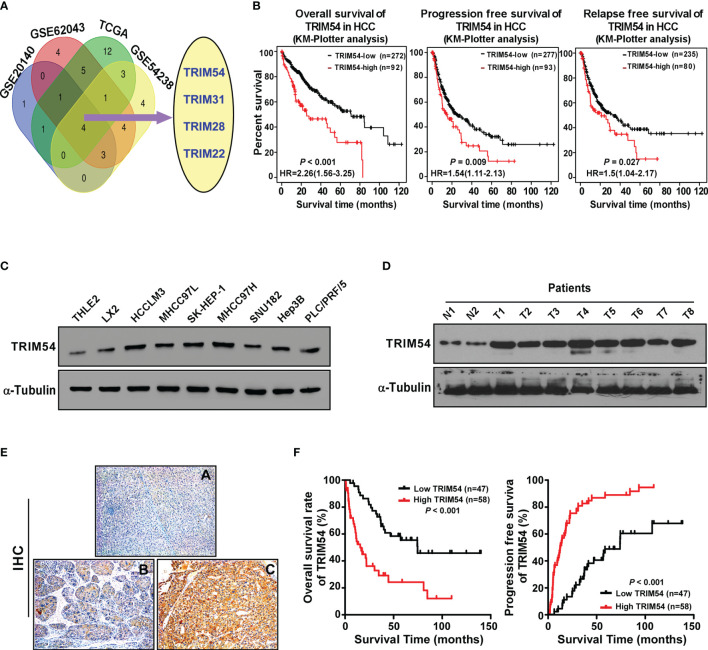
TRIM54 overexpression correlates with progression and poor prognosis in hepatocellular carcinoma. **(A)** Expression profiling of TRIM54 mRNAs was studied by using VENN diagram. **(B)** Kaplan-Meier analysis of overall (left) or progression-free (middle) or relapse-free (right)survival curves from public dataset for HCC patients with low TRIM54 expression or high TRIM54 expression. **(C)** Western blot analysis of TRIM54 in in 2 normal liver cells and in 7 HCC cells. α-Tubulin was used as a loading control. **(D)** Western blotting analysis of TRIM54 expression in 2 non-tumor tissues (N) and in 8 HCC tissues. α-Tubulin was used as a loading control. **(E)** IHC staining indicating the TRIM54 protein expression in normal tissues **(A)** and HCC **(B, C)**. **(F)** The Kaplan-Meier survival curves compare HCC patients with low and high TRIM54 expression levels, P < 0.001.

In order to identify the important TRIM protein molecules that are involved in the progression of HCC, we analyze the mRNA expression of approximately 70 known TRIM proteins in multiple published profiles including TCGA, GSE54238, GSE20140, and GSE62043. By using the Venn diagram approach, we found the four TRIM family proteins (TRIM54, TRIM31, TRIM28, TRIM22) which showed significant changes in all four datasets. Among them (**Figure 1A**), TRIM54, TRIM31, and TRIM28 showed significant overexpression in GSE54238 (advanced HCC vs. early HCC > 1.5, *p* < 0.05), TCGA (HCC vs. normal > 1.5, *p* < 0.05), and GSE20140 (HCC tissues vs. chronic tissues > 1.5, *p* < 0.05) and an mRNA expression of the top 20 TRIM proteins in 100 primaries and matched non-malignant tissues of HCC patients; however, TRIM22 was downregulated in the above multiple published profiles (T/N<0.5, *p* < 0.05). Further survival analysis of the four TRIM proteins in HCC patients (http://kmplot.com/analysis/index.php?p=service&cancer=liver_rnaseq) showed that a higher TRIM54 expression was associated not only with a shorter overall survival time (HR=2.26, *p* < 0.001) but also with progression-free survival time (HR=1.54, *P* = 0.009) and relapse-free survival time (HR=1.50, *P* = 0.027) compared with a lower TRIM54 expression in HCC patients (**Figure 1B**), but not TRIM31 and TRIM22 (**Supplemental Figure 1A**). The mRNA expression of TRIM28 was also associated with overall survival and progression-free survival time (**Supplemental Figure 1A**), although it has been reported in HCC previously; therefore, we focus on the biological function and molecular mechanism of TRIM54 in HCC in the current study. Interestingly, real-time PCR and Western blotting analyses revealed that TRIM54 was significantly overexpressed in HCC cell lines and HCC tissues at both protein and mRNA levels, compared with the two normal liver cells and two non-tumor specimens (**Figures 1C, D** and **Supplemental Figures 1B, C**), suggesting that TRIM54 is upregulated in hepatocellular carcinoma tissues.

Next, TRIM54 expression was further examined in 105 archived HCC tissues by IHC assay to analyze the clinical relevance of TRIM54 in HCC. As shown in **Figure 1D** and **Supplemental Tables 1, 2**, TRIM54 levels were correlated with the pathologic TNM (*p* = 0.01) in HCC patients. The increased expression of TRIM54 was detected in the clinical hepatocellular carcinoma tissue samples, but not detectable in adjacent normal liver tissue from the same HCC patient (**Figure 1E**). Importantly, statistical analysis showed that hepatocellular carcinoma patients with a high TRIM54 expression had a significantly worse overall survival and higher recurrent rate than those with a low TRIM54 expression (**Figure 1F**). These results suggest that TRIM54 has potential clinical value as a predictive biomarker for disease outcome in hepatocellular carcinoma.

### Ectopic Expression of TRIM54 Promoted HCC Proliferation and Metastasis *In Vivo*

**Figure 2:**
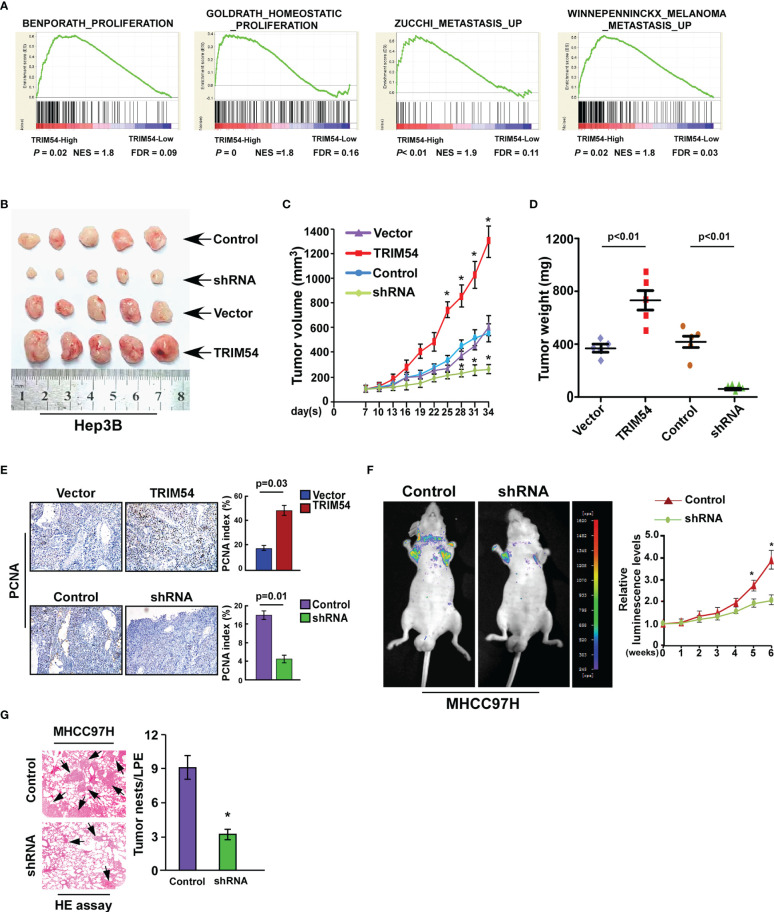
Ectopic expression of TRIM54 promoted HCC proliferation and metastasis in vivo. **(A)** GSEA plot, indicating a significant correlation between the mRNA levels of TRIM54 and the proliferation and metastasis gene signatures in published datasets. **(B)** Images of the tumors from indicated mice in each group (subcutaneous injection). **(C)** Tumor volumes were measured on the indicated days. **(D)** Mean tumor weights. **(E)** The IHC staining indicating the PCNA protein expression in the indicated group. *P < 0.05. **(F)** Tail vein injection model examined the lung metastatic ability of MHCC97H-Control and MHCC97H -shTRIM54 cells (n = 5) (left), quantification of bioluminescence signal was measured on the indicated days (right). *P < 0.05. **(G)** The HE staining indicating the tumor nests in the indicated group. *P < 0.05. Each bar represents the mean ± SD of three independent.

We next explore the biological function of TRIM54 in HCC; gene set enrichment analysis (GSEA) revealed that TRIM54 overexpression significantly correlated with gene signatures associated with proliferation and metastasis in the TCGA dataset of HCC, suggesting that TRIM54 might contribute to growth and metastasis in HCC (**Figure 2A**). To investigate the pro-growth and pro-metastasis roles of TRIM54 in HCC progression, we used the Hep3B cell line with a medium level of TRIM54 expression for both knockdown and overexpression modifications, the knockdown experiment in TRIM54 highly expressing cells, which gradually increases metastatic potential (MHCC97H), and the overexpression experiment in the cells with low TRIM54 expression (SUN182) (**Supplemental Figure 2**). A subcutaneous xenografted tumor model was firstly used to examine the biological function of TRIM54 in HCC progression *in vivo*. As shown in **Figures 2B–D**, the tumors formed by Hep3B/TRIM54-overexpression cells were larger in both volume and weight than the tumors formed by control cells. Conversely, the tumors formed by Hep3B/TRIM54-shRNA cells were smaller and lighter than the control tumors. IHC analysis revealed that TRIM54-overexpressed tumors showed higher percentages of PCNA-positive cells, whereas TRIM54-inhibition tumors displayed lower percentages of PCNA-positive cells than the control tumors (**Figure 2E**). Furthermore, the tail vein injection model was used to examine the pro-metastasis potential of TRIM54 in HCC. Interestingly, we found that inhibition of TRIM54 significantly represses the distant lung metastasis of MHCC97H cell line (**Figures 2F, G**). Collectively, these results demonstrate that TRIM54 functions as a tumor oncogenic gene in HCC *in vivo*.

### Ectopic Expression of TRIM54 Promoted HCC Proliferation and Metastasis *In Vitro*

**Figure 3:**
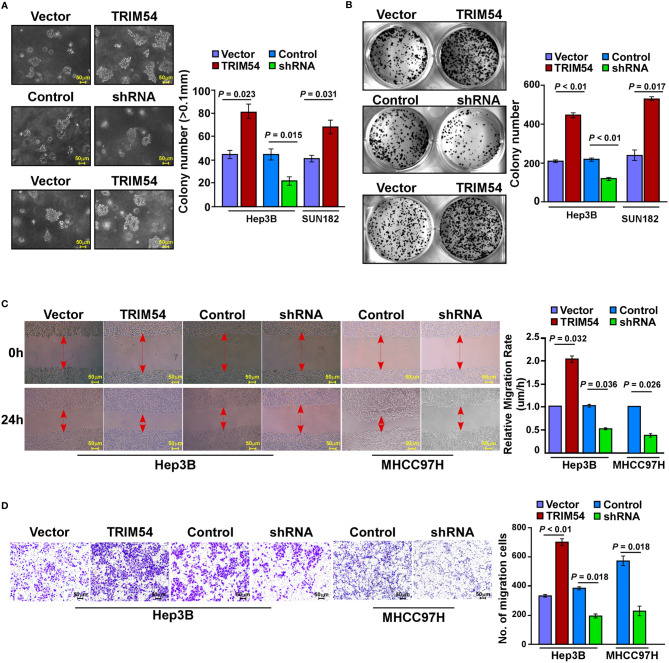
Ectopic expression of TRIM54 promoted HCC proliferation and metastasis in vitro. **(A)** Representative micrographs (left) and quantification of colonies > 0.1 mm (right) were scored. Indicated cells (2 × 103) were suspended in soft agar and cultured for 10 days, and then colonies > 0.1 mm in diameter were counted. **(B)** Representative micrographs (left) and quantification (right) of crystal violet-stained cell colonies. Indicated cells (0.8 × 103) were plated into six-well plates and cultured for 10 days, then stained with crystal violet (1.0%). **(C)** Representative micrographs of wound healing assay of the indicated cells. Wound closures were photographed at 0 and 24 hours after wounding. **(D)** Quantification of indicated migration cells in ten random fields analyzed by transwell assays, respectively. Each bar represents the mean ± SD of three independent experiments.

We further examined the effect of TRIM54 on HCC cell proliferation and metastasis *in vitro*. An anchorage-independent growth assay revealed that Hep3B and SUN182 cells stably expressing TRIM54 showed more and larger-sized colonies than control cells; however, TRIM54 suppression dramatically decreased the growth rate of HCC cells compared with that of control cells (**Figure 3A**). In addition, colony formation assay showed that overexpression of TRIM54 significantly increased, but inhibition of TRIM54 repressed, the growth rate of HCC cells compared with that of control cells (**Figure 3B**). Furthermore, TRIM54-overexpressing cells increased but downregulation of TRIM54 inhibited the metastatic capacity compared with the control cells, as examined by wound heading and transwell assays (**Figures 3C, D**). These results indicated that TRIM54 is involved in the regulation of the pro-growth and pro-metastasis capability process of HCC cells *in vitro*.

### Overexpression of TRIM54 Sustains wnt/β-Catenin Activity

**Figure 4:**
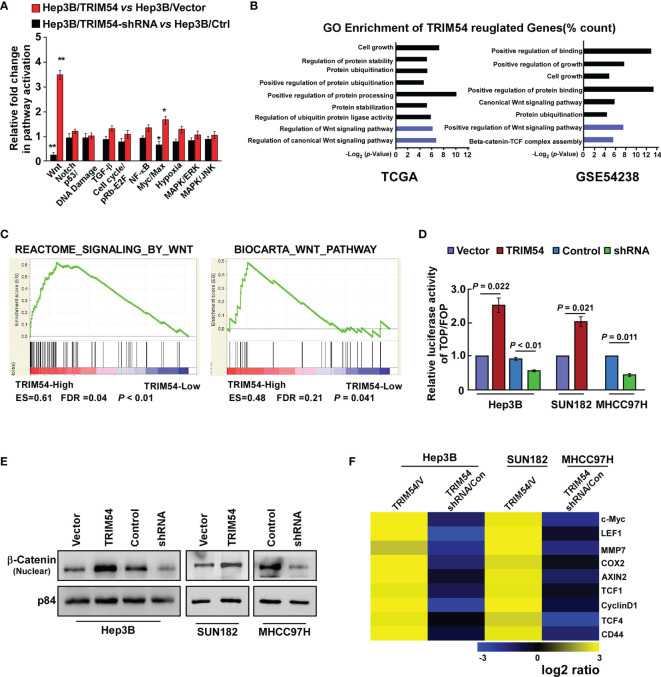
Overexpression of TRIM54 sustains wnt/β-catenin activity. **(A)** Cignal finder reporter arrays showing that overexpression of TRIM54 significantly activated wnt/β-catenin signaling in the indicated cells, **p < 0.01, *p < 0.05. **(B)** GO enrichment analysis of the TRIM54 regulated-transcripts identified in a published gene set (TCGA and GSE54238). **(C)** GSEA analysis showing that *TRIM54* mRNA levels were correlated with wnt/β-catenin-related gene signature in the TCGA HCC dataset. **(D)** Indicated cells transfected with TOPflash or FOPflash and Renilla pRL-TK plasmids were subjected to dual-luciferase assays 48 h after transfection. Reporter activity detected was normalized by Renilla luciferase activity. **(E)** Nuclear fractions of β-catenin in indicated cells were analyzed by western blotting; p84 was used as the loading control. **(F)** Real-time PCR analysis indicating an apparent overlap between wnt/β-catenin-dependent gene expression and TRIM54-regulated gene expression. The pseudocolor represents the intensity scale of TRIM54 versus the vector, or TRIM54-shRNA versus the control, generated by a log2 transformation. Each bar represents the mean ± SD of three independent experiments.

The molecular mechanism of TRIM54-promoted tumorigenesis and metastasis on HCC was further examined. Cignal Finder reporter arrays revealed that the overexpression of TRIM54 in Hep3B cells resulted in significant wnt/β-catenin activation; however, inhibition of TRIM54 in Hep3B cells repressed the wnt/β-catenin activity (**Figure 4A**). Gene ontology enrichment analysis revealed that genes with GO biological process terms “Regulation of Wnt signaling pathway,” “Regulation of Wnt signaling pathway,” “Positive regulation of Wnt signaling pathway,” and “Beta-catenin-TCF complex assembly” were enriched (**Figure 4B**). The correlation between TRIM54 expression and wnt/β-catenin activation was further determined by using gene set enrichment analysis (GSEA), which showed that TRIM54 overexpression was significantly associated with gene signature-related wnt/β-catenin activation in HCC (**Figure 4C**), suggesting that TRIM54 might contribute to modulate wnt/β-catenin signaling. This hypothesis was further confirmed by multiple assays, in which overexpression of TRIM54 significantly increased, but inhibition of TRIM54 reduced wnt/β-catenin-driven luciferase activity, nuclear β-catenin nuclear expression, and expression of numerous well-known wnt/β-catenin targets (**Figures 4D–F**), demonstrating that the TRIM54 overexpression sustains wnt/β-catenin activity.

### TRIM54 Activates wnt/β-Catenin Signaling *via* Ubiquitylation and Destabilization of Axin1

**Figure 5:**
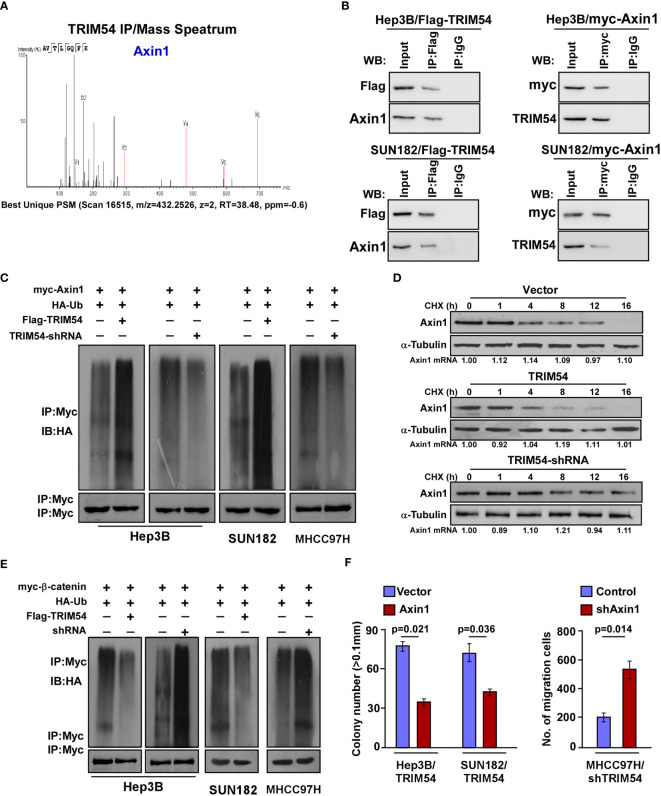
TRIM54 activates wnt/β-catenin signaling *via* ubiquitylating and destabilizing of Axin1. **(A)** Representative mass spectrometry plots and sequences of peptides from Axin1. **(B)** Co-IP assay showing that endogenous TRIM54 interacted with endogenous Axin1 in Hep3B cells. **(C)** Western blotting analysis of the polyubiquitin levels of Axin1 in the indicated cells. **(D)** Western blotting analysis of the half-life of Axin1 protein in the indicated cells. a-Tubulin served as a loading control. **(E)** Western blotting analysis of the polyubiquitin levels of β-catenin in the indicated cells. **(F)** Relative colony number (left) and migration rate (right) in the indicated cells. Each bar represents the mean ± SD of three independent experiments.

We next preformed affinity purification/mass spectrometry (IP/MS) to identify potent TRIM54-binding proteins that activate wnt/β-catenin activity. As shown in **Figure 5A**, IP/MS analysis demonstrates that Axin1, which functions as a negative regulator of the wnt/β-catenin signaling pathway, was the potent TRIM54-binding protein. Overexpression of Axin1 significantly decreased the TOP/FOP luciferase activity in TRIM54-overexpression cell-induced β-catenin signaling activation, but inhibition of Axin1 significantly increased the TOP/FOP luciferase activity in TRIM54-shRNA cell-induced β-catenin signaling activation (**Supplemental Figure 3**). Co-immunoprecipitation (co-IP) analyses demonstrated that TRIM54 directly interacts with Axin1 (**Figure 5B**). TRIM54 acted as a TRIM protein family containing a RING finger motif, which plays an important role in ubiquitination modification. Consistent with the E3 ubiquitin ligase function, overexpression of TRIM54 increased, but inhibition of TRIM54 decreased the level of the polyubiquitination modification of Axin1 protein in HCC cell lines (**Figure 5C**). Moreover, the half-life level of Axin1 protein was decreased in TRIM54 overexpression; however, it increased in the inhibition of TRIM54 cell lines but had no effect on its mRNA expression (**Figure 5D**). In addition, the ubiquitination of β-catenin was reduced by TRIM54 overexpression but increased by TRIM54 knockdown in HCC cells (**Figure 5E**). Consistently, the promoting effects of TRIM54 overexpression on growth and metastasis were drastically reduced by silencing Axin1 (**Figure 5F**). These results suggest TRIM54 pro-growth and pro-metastasis *via* triggering of β-catenin signaling through Axin1 degradation in HCC cells.

### Wnt/β-Catenin Signaling Pathway Is Required for TRIM54-Induced Pro-Proliferation and Pro-Metastasis Ability on Human HCC

**Figure 6:**
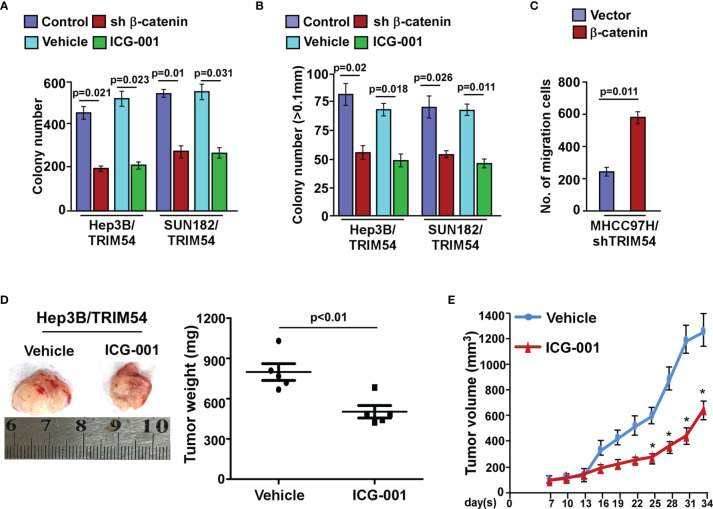
Wnt/β-catenin signaling pathway is required for TRIM54 induced pro-proliferation and pro-metastasis ability on human HCC. **(A)** Quantification of colony numbers in indicated HCC cells, as determined by colony formation. **(B)** Quantification of colonies > 0.1 mm in indicated HCC cells, as determined by soft agar assay. **(C)** Number of invaded cells in indicated HCC cells. **(D)** Images of the tumors from indicated mice in each group (left); mean tumor weights (right). **(E)** IHC staining demonstrated the expression of PCNA-positive cells in the indicated tissues, *p < 0.05. Each bar represents the mean ± SD of three independent experiments.

Next, we investigated whether TRIM54 mediated HCC proliferation and metastasis through Wnt/β-catenin activation. The proliferation and metastasis effect of TRIM54 on HCC through Wnt/β-catenin activation was determined by anchorage-independent growth assay, colony formation, and invasive assay. Strikingly, we found that blockade of the Wnt/β-catenin pathway by β-catenin siRNA significantly abrogates the effect of TRIM54 on HCC aggressiveness *in vitro* (**Figures 6A–C**). Similar to the effect of β-catenin silencing in TRIM54 overexpression cells, treatment with ICG-001, a specific inhibitor of β-catenin signaling *via* blockage of β-catenin/CBP interaction, also significantly decreased the effect of TRIM54 on HCC proliferation and metastasis (**Figures 6A–C**). Interestingly, treatment with a WNT inhibitor (ICG-001) significantly inhibited the pro-growth effects of TRIM54 in HCC *in vivo* (**Figures 6D, E**). Thus, the above results indicate that the activation of the Wnt/β-catenin signaling pathway plays important effects of TRIM54 on HCC aggressiveness.

### Clinical Relevance of TRIM54-Induced Wnt/β-Catenin Activation in Human HCC

**Figure 7:**
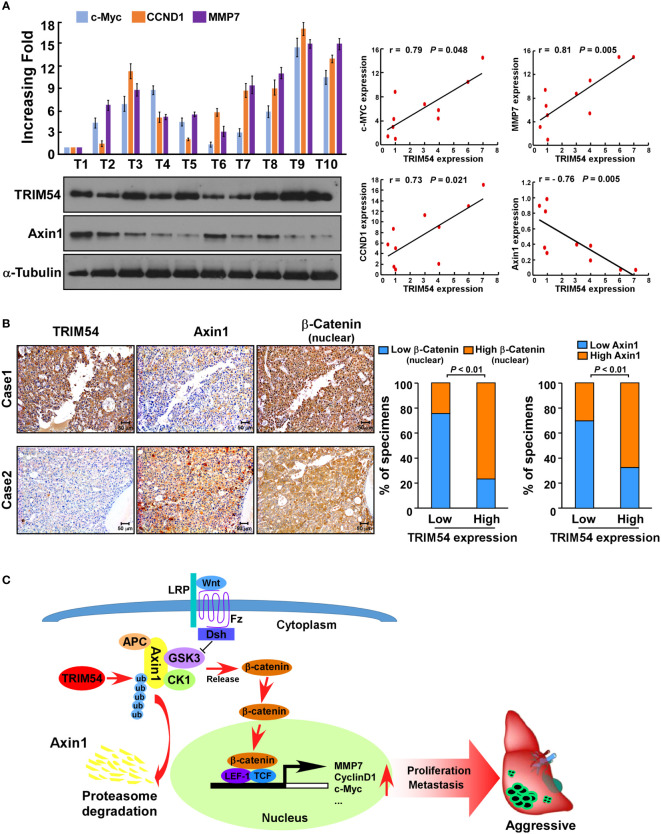
Clinical relevance of TRIM54 and the expression of β-catenin of Axin1 in HCC. **(A)** Analysis of expression (left) and correlation (right) of TRIM54 with MMP7, c-MYC, and CCND1 mRNA expression, as well as the protein levels of Axin1 in 10 freshly collected HCC samples. **(B)** TRIM54 levels were positively associated with nuclear β-catenin expression in 105 primary human HCC specimens. Two representative cases are shown. p < 0.01. **(C)** Schematic diagram illustrating that overexpression of TRIM54 facilitates proliferation and metastasis and activating the Wnt/β-catenin pathway by ubiquitous degradation of Axin1 in hepatocellular carcinoma. Each bar represents the mean ± SD of three independent experiments.

Finally, we examined whether the TRIM54/Axin1/β-catenin axis identified in HCC cell models could be also verified in clinical HCC tumors. Western blot assay showed that TRIM54 was inversely correlated with the expression levels of Axin1(r=-0.76, *p* = 0.005) and significantly correlated with the mRNA levels of c-myc (r=0.79, *p* = 0.048), CCND1 (r=0.73, *p* = 0.021), and MMP7 (r=0.81, *p* = 0.005) in 10 freshly collected clinical HCC samples (**Figure 7A**). Consistently, analysis of 105 HCC tissue specimens using IHC analysis showed that TRIM54 expression was correlated with the expression levels of nuclear β-catenin (*p* < 0.01) and showed an inverse correlation with the expression levels of Axin1 (*p* < 0.01) (**Figure 7B**). These data further supported the notion that overexpression of TRIM54 in HCC reduces Axin1 expression and activates Wnt/β-catenin signaling, ultimately leading to tumorigenesis and metastasis and poor clinical outcomes for human HCC (**Figure 7C**).

## Discussion

TRIM54, also known as MURF, was an identified RING-type E3 ubiquitin-protein ligase and a myogenic regulator of skeletal myoblast differentiation and myotube fusion (22–24). By function as an E3 ubiquitin ligase, TRIM54 was demonstrated to be involved in the development and early postnatal adaptation of skeletal muscle in ubiquitin-mediated muscle protein turnover (25). It is also reported that TRIM54 was a novel and sensitive biomarker for the diagnosis of acute myocardial infarction, and heterozygous TRIM54 mutation may contribute to cardiac and skeletal protein aggregate myopathy (26, 27). These studies suggest a pivotal role of TRIM54 in various pathological processes. However, the clinical significance and biological role of TRIM54 in carcinogenesis remain largely unknown. In this study, by analyzing multiple published mRNA expression profiles, we found that the expression of TRIM54 was significantly higher in all the four datasets. Further analysis of TCGA datasets and our HCC tissue samples showed that TRIM54 was associated with overall survival and progression-free survival in HCC patients. *In vitro* and *in vivo* experiments demonstrate that TRIM54 overexpression in tumor cells facilitates tumorigenesis and intrahepatic metastasis; however, inhibition of TRIM54 significantly suppressed hepatocellular carcinoma progression, which indicates that TRIM54 may be a promising prognostic biomarker for HCC.

Axis inhibition protein 1 (Axin1) is an important negative regulator of the Wnt/β-catenin cascade by forming a cytoplasmic phosphorylated destruction complex with adenomatous polyposis coli (APC), glycogen synthase kinase-3 beta (GSK3β), and casein kinase-1 (28, 29). It is reported that constitutive activation of Wnt/β-catenin signaling is achieved mainly by downregulation of protein levels of negative regulators, such as the AXIN genes. However, loss-of-function mutations of AXIN are less frequent, with occurrence rates of ~5%–9% of human HCC samples (30). However, β-catenin nuclear accumulation has been observed in more than 50% of HCC tumors (31), which suggested that there are other mechanisms involved in the downregulation of AXIN protein levels in HCC. In our study, by using affinity purification/mass spectrometry (IP/MS) and co-immunoprecipitation (co-IP) analyses, we found that TRIM54 directly interacts with Axin1. Furthermore, the level of the polyubiquitination modification of Axin1 protein was increased and the half-life level of Axin1 protein was decreased in TRIM54 overexpression HCC cells. These results suggest that overexpression of TRIM54 was contributed to Axin1 protein degradation in HCC cells.

Hyperactivation of the Wnt/β-catenin cascade is one of the most frequent molecular events in various cancers, and activation of this pathway is thought to be an early event in tumorigenesis (32, 33). It is reported that the mutation rates of the β-catenin gene are only 12%–25% in HCCs and APC and AXIN mutations are less frequent (34, 35). However, nuclear accumulation of β-catenin is occurring in 40%–70% of HCCs (36), which suggest that there are other critical regulators involved in activation of the Wnt/β-catenin pathway in HCC. In the current study, by performing Cignal Finder reporter arrays, we found that the overexpression of TRIM54 in HCC cells resulted in significant wnt/β-catenin activation. Gene ontology enrichment analysis and biological experiment further confirmed that overexpression of TRIM54 sustaining wnt/β-catenin activity in HCC. The above studies suggested that TRIM54 may represent an important target for clinical intervention in HCC by controlling Wnt/β-catenin signaling.

## Data Availability Statement

The datasets presented in this study can be found in online repositories. The names of the repository/repositories and accession number(s) can be found in the article/**Supplementary Material**.

## Ethics Statement

The studies involving human participants were reviewed and approved by the Institutional Research Ethics Committee of Taizhou Hospital of Zhejiang Province. The patients/participants provided their written informed consent to participate in this study. The animal study was reviewed and approved by the Institutional Animal Care and Use Committee of Guangdong Pharmaceutical University.

## Author Contributions

JZ and RZ designed all experiments, supervised the project, and wrote the manuscript. YW performed all the immunoprecipitation experiments and conducted the MS analysis. YY and JS provided patient tissue samples and analyzed clinical data. JZ and SL established the expressing plasmid and conducted the luciferase assay. JZ and NZ established the real time-PCR analysis. JZ, CF and YL performed the in vivo experiments and conducted the IHC assay. All authors contributed to the article and approved the submitted version.

## Funding

This work was supported by the Science and Technology Department of Guangdong Province (No. 2019A1515110740) and Health Commission of Guangdong Province (No. A2020100).

## Conflict of Interest

The authors declare that the research was conducted in the absence of any commercial or financial relationships that could be construed as a potential conflict of interest.

## Publisher’s Note

All claims expressed in this article are solely those of the authors and do not necessarily represent those of their affiliated organizations, or those of the publisher, the editors and the reviewers. Any product that may be evaluated in this article, or claim that may be made by its manufacturer, is not guaranteed or endorsed by the publisher.
