# Supplementary material for: Targeting TRIM54/Axin1/β-Catenin Axis Prohibits Proliferation and Metastasis in Hepatocellular Carcinoma
Source: Front Oncol. 2021 Dec 9;11:759842. doi: 10.3389/fonc.2021.759842 (PMC8695909; doi:10.3389/fonc.2021.759842)
Supplement: Supplementary file 1 [file DataSheet_1.doc]

**Supplementary Information**

**Supplemental Figure Legends**

**Supplemental Figure** 1. (A). Kaplan-Meier analysis of public dataset for HCC patients. (B). Real-time PCR analysis of TRIM54 expression in 2 normal liver cells and in 7 HCC cells. Transcript levels were normalized to GAPDH expression. (C). Real-time PCR analysis of TRIM54 expression in 2 non-tumor tissues (N) and in 8 HCC tissues. Transcript levels were normalized to GAPDH expression. Each bar represents the mean ± SD of three independent experiments.

**Supplemental Figure** 2. Western blot analysis of TRIM54 in the indicated HCC cells. α-Tubulin was used as a loading control.

**Supplemental Figure** 3. Indicated cells transfected with TOPflash or FOPflash and Renilla pRL-TK plasmids were subjected to dual-luciferase assays 48 hours after transfection. Reporter activity detected was normalized by Renilla luciferase activity. Each bar represents the mean ± SD of three independent experiments. * *P* < 0.05.

**Supplemental Table 1. The relationship of TRIM54 expression in 105 HCC with clinical pathological characteristics.**

|  |  |  | TRIM54 expression | |  |
| --- | --- | --- | --- | --- | --- |
| Characteristics | Subgroup | Patients, Number(%) | Low(47) | High(58) | *P* values |
| Gender |  |  |  |  |  |
|  | Male | 85(80.9) | 37 | 48 | 0.626 |
|  | Female | 20(19.1) | 10 | 10 |  |
| Age (years) |  |  |  |  |  |
|  | ≤50 | 56(53.3) | 25 | 31 | 0.567 |
|  | >50 | 49(46.7) | 22 | 27 |  |
| HBsAg |  |  |  |  |  |
|  | Negative | 18(17.1) | 15 | 13 | 0.375 |
|  | Positive | 87(82.9) | 32 | 45 |  |
| AFP, ng/mL |  |  |  |  |  |
|  | ≤20 | 45(42.9) | 25 | 20 | 0.074 |
|  | >20 | 60(57.1) | 22 | 38 |  |
| Liver cirrhosis |  |  |  |  |  |
|  | Absent | 16(15.2) | 8 | 8 | 0.786 |
|  | Present | 89(84.8) | 39 | 50 |  |
| Tumor size, cm |  |  |  |  |  |
|  | ≤5 | 46(43.8) | 23 | 23 | 0.429 |
|  | >5 | 59(56.2) | 24 | 35 |  |
| Pathologic TNM |  |  |  |  |  |
|  | I/II | 69(65.7) | 37 | 32 | 0.014***** |
|  | III/IV | 36(34.3) | 10 | 26 |  |

**Supplemental Table 2. Univariate and multivariate analysis of different prognostic parameters in patients with HCC by Cox-regression analysis**

|  | **Univariate analysis** | | **Multivariate analysis** | |
| --- | --- | --- | --- | --- |
|  | ***P*** | **Hazard ratio**  **(95% CI)** | ***P*** | **Hazard ratio**  **(95% CI)** |
| **Gender** | 0.691 | 0.889  (0.498-1.586) | 0.745 | 0.903  (0.489-1.669) |
| Male |  |  |  |  |
| Female |  |  |  |  |
| **Age (years)** | 0.705 | 1.014  (0.738-1.616) | 0.310 | 0.599  (0.223-1.609) |
| ≤50 |  |  |  |  |
| >50 |  |  |  |  |
| **HBsAg** | 0.032 | 1.553  (1.100-2.345) | 0.713 | 0.895  (0.496-1.617) |
| Negative |  |  |  |  |
| Positive |  |  |  |  |
| **AFP, ng/mL** | 0.334 | 1.267  (0.784-2.046) | 0.076 | 1.596  (0.952-2.674) |
| ≤20 |  |  |  |  |
| >20 |  |  |  |  |
| **Liver cirrhosis** | 0.047 | 1.219  (1.040-2.469) | 0.208 | 1.455  (0.811-2.609) |
| Absent |  |  |  |  |
| Present |  |  |  |  |
| **Tumor size, cm** | 0.795 | 1.066  (0.660-1.721) | 0.863 | 0.957  (0.580-1.578) |
| ≤5 |  |  |  |  |
| >5 |  |  |  |  |
| **Pathologic** **TNM** | 0.025 | 2.339  (1.113-4.914) | 0.010 | 2.929  (1.300-6.600) |
| I/II |  |  |  |  |
| III/IV |  |  |  |  |
| **TRIM54 level** | < 0.001 | 4.372  (1.994-9.585) | 0.002 | 5.247  (1.798-15.310) |
| Low expression |  |  |  |  |
| High expression |  |  |  |  |

**Supplemental Tables 3 Primers and Oligonucleotides used in this study.**

| **Used for subcloning and plasmid construction:** | |
| --- | --- |
| **Primer used for FLAG tagged TRIM54** | |
| **TRIM54-forwards** | gccTCTAGAgccatgGATTACAAGGACGACGATGACAAGaacttcacagtgggttt |
| **TRIM54-downwards** | gccGGATCCttaaggcccatccggccgct |
| **Primer used for qPCR** | |
| TRIM54-up | ATCGTGCAGGCATGAGGTTG |
| TRIM54-dn | CCTCGCACATGAGGTGCTG |
| c-MYC-up | GGCTCCTGGCAAAAGGTCA |
| c-MYC-dn | CTGCGTAGTTGTGCTGATGT |
| LEF1-up | AGAACACCCCGATGACGGA |
| LEF1-dn | GGCATCATTATGTACCCGGAAT |
| MMP7-up | GAGTGAGCTACAGTGGGAACA |
| MMP7-dn | CTATGACGCGGGAGTTTAACAT |
| COX2-up | CCTGTATGCCCTTTTCCTAA |
| COX2-dn | GATCGTTGACCTCGTCTGTT |
| AXIN2-up | CAACACCAGGCGGAACGAA |
| AXIN2-dn | GCCCAATAAGGAGTGTAAGGACT |
| TCF1-up | AACACCTCAACAAGGGCACTC |
| TCF1-dn | CCCCACTTGAAACGGTTCCT |
| CCND1-up | GCTGCGAAGTGGAAACCATC |
| CCND1-dn | CCTCCTTCTGCACACATTTGAA |
| TCF4-up | CAAGCACTGCCGACTACAATA |
| TCF4-dn | CCAGGCTGATTCATCCCACTG |
| CD44-up | CTGCCGCTTTGCAGGTGTA |
| CD44-dn | CATTGTGGGCAAGGTGCTATT |
| **Oligonucleotides for shRNAs** | |
| TRIM54 shRNA | CCGGGCCAGACTATCGAGGACAATACTCGAGTATTGTCCTCGATAGTCTGGCTTTTTG |
| β-catenin shRNA | CCGGCCCGTGTAAATATGTACATTTCTCGAGAAATGTACATATTTACACGGGTTTTTG |
| AXIN1 shRNA | CCGGAGGTGCTATCTGTCTGCTCTACTCGAGTAGAGCAGACAGATAGCACCTTTTTT |
