# Supplementary figures and images for: Targeting TRIM54/Axin1/β-Catenin Axis Prohibits Proliferation and Metastasis in Hepatocellular Carcinoma
Source: Front Oncol. 2021 Dec 9;11:759842. doi: 10.3389/fonc.2021.759842 (PMC8695909; doi:10.3389/fonc.2021.759842)

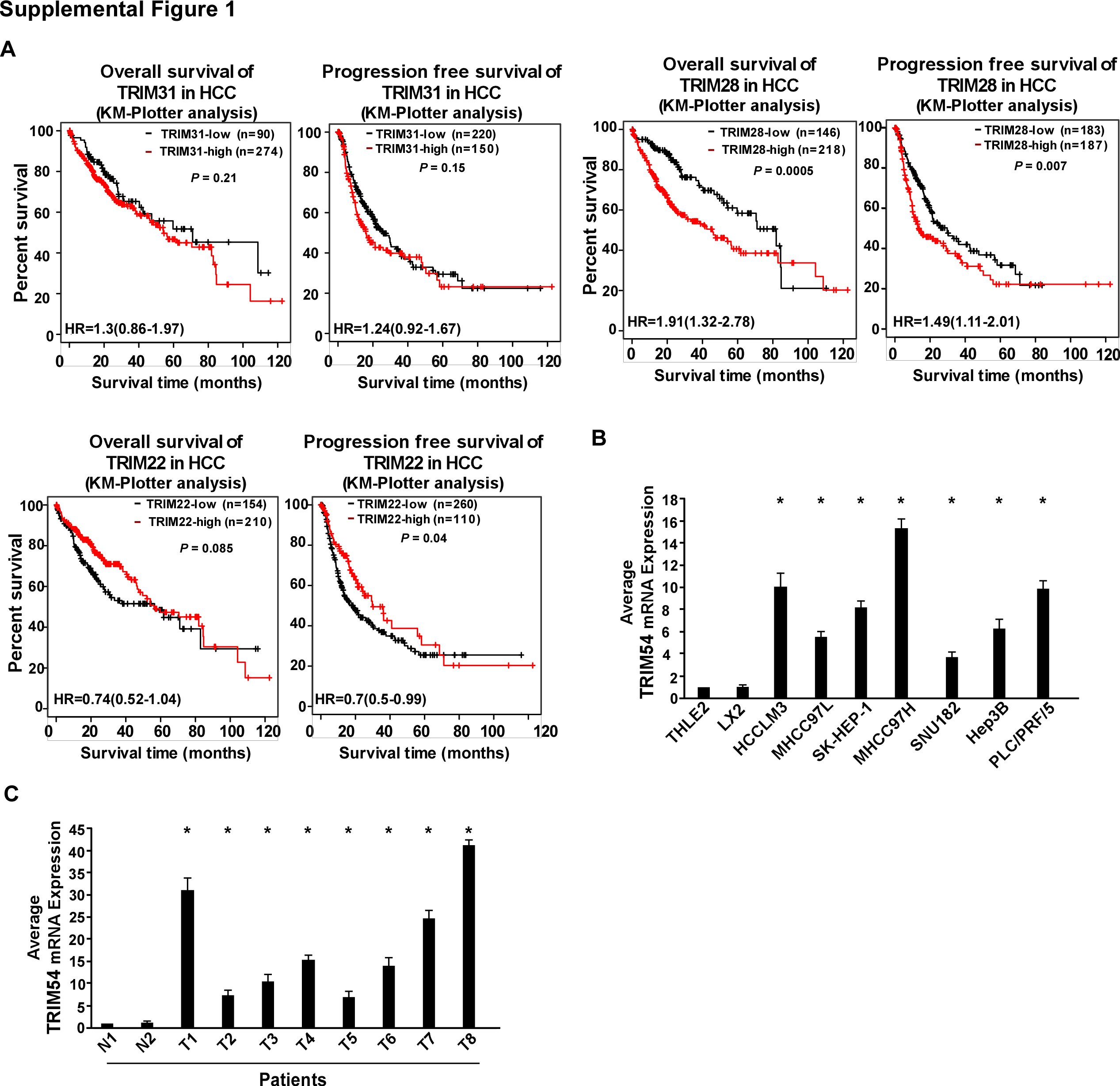

Supplement: Supplementary file 3 [file Image_1.tif]

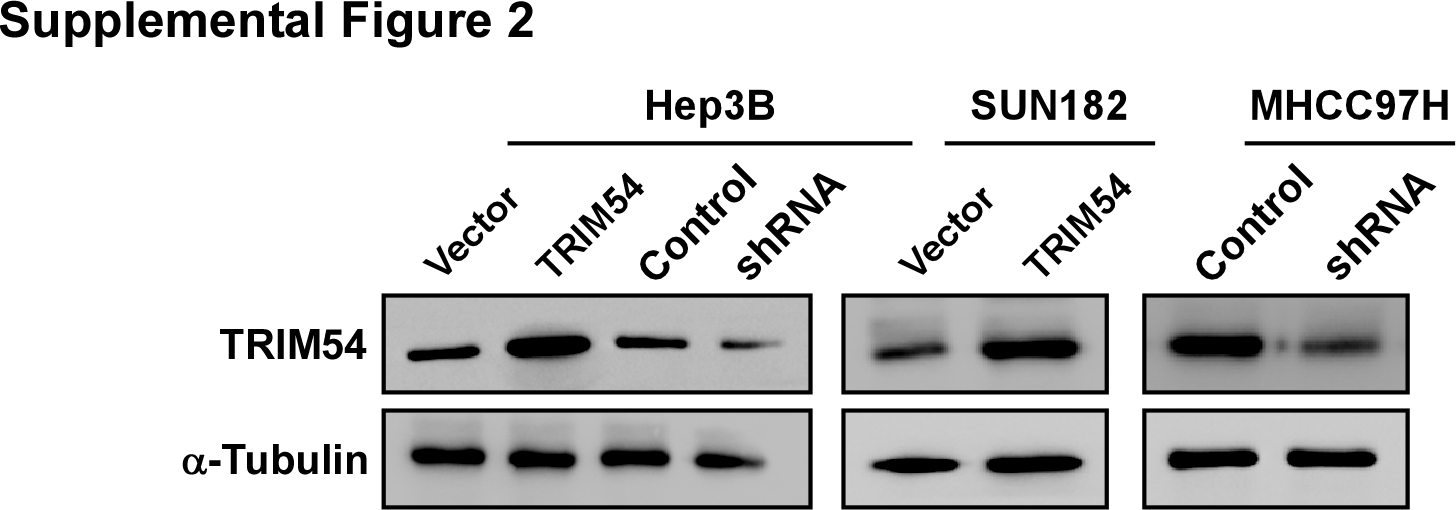

Supplement: Supplementary file 4 [file Image_2.tif]

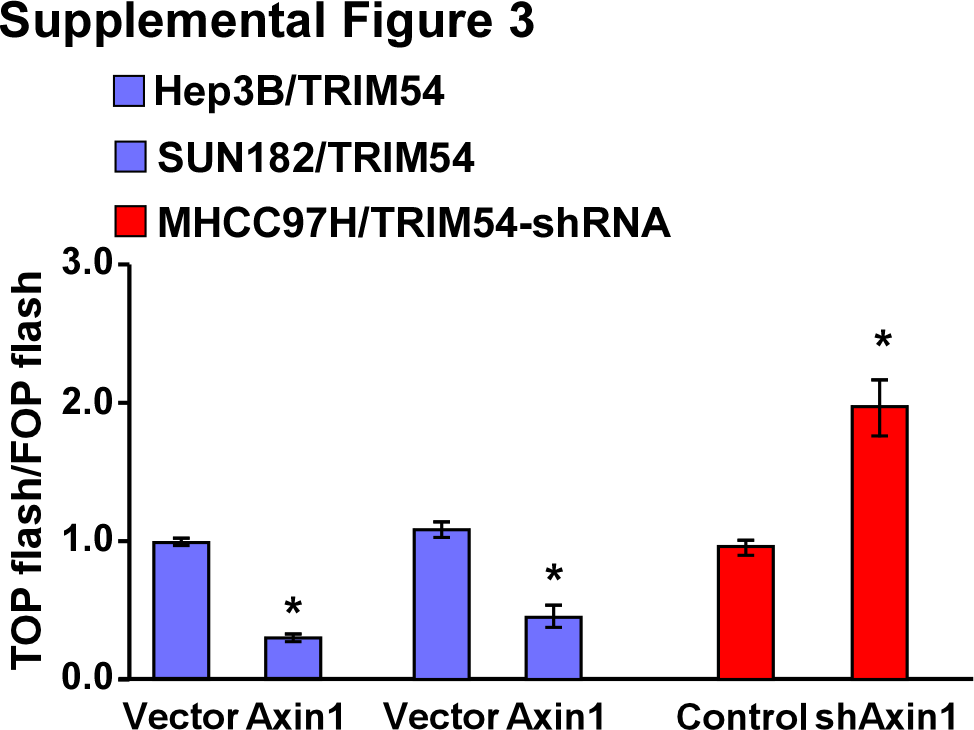

Supplement: Supplementary file 5 [file Image_3.tif]

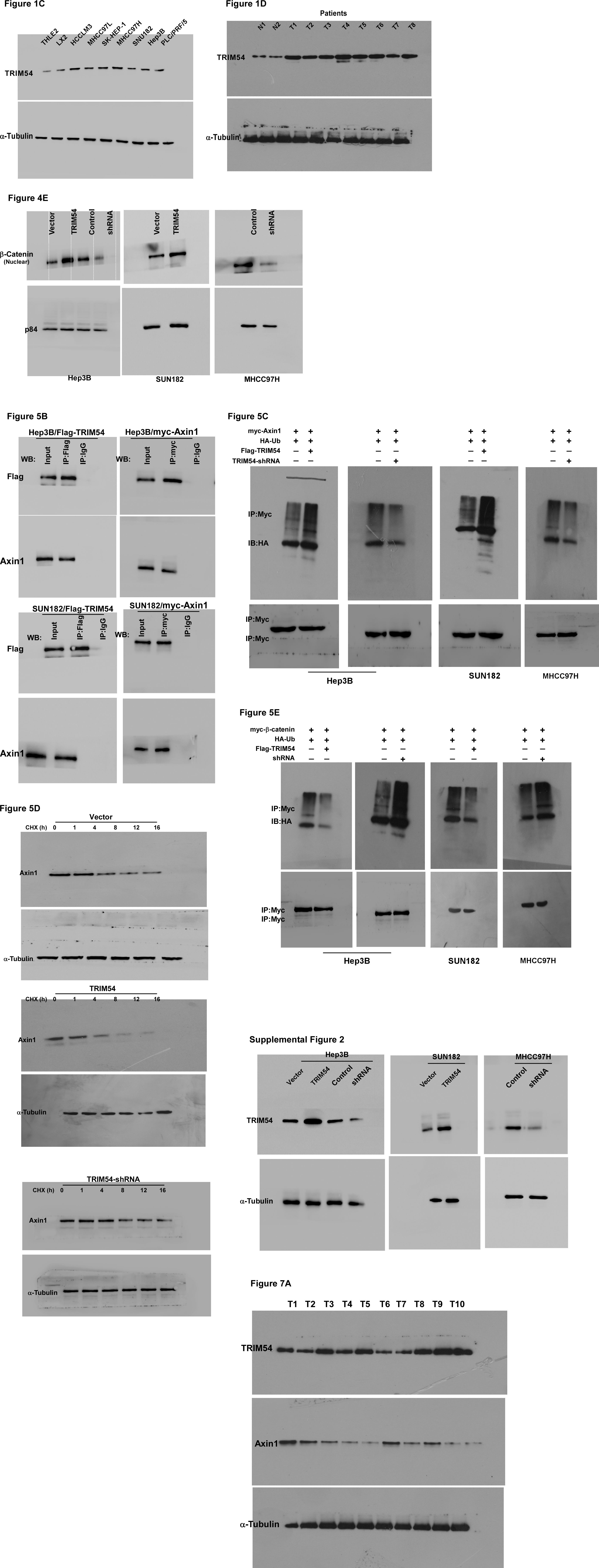

Supplement: Supplementary file 6 [file Image_4.tif]

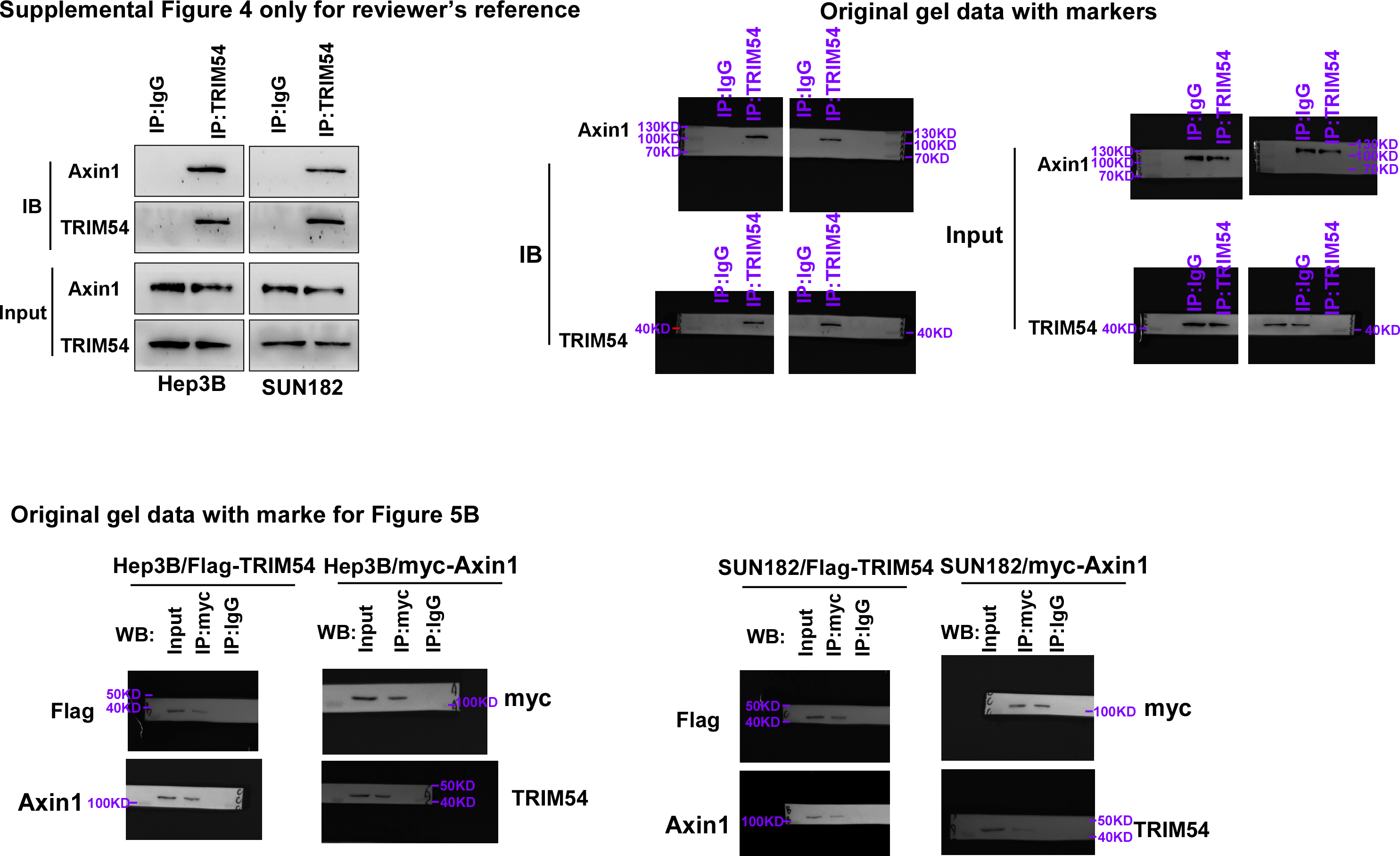

Supplement: Supplementary file 7 [file Image_5.tif]

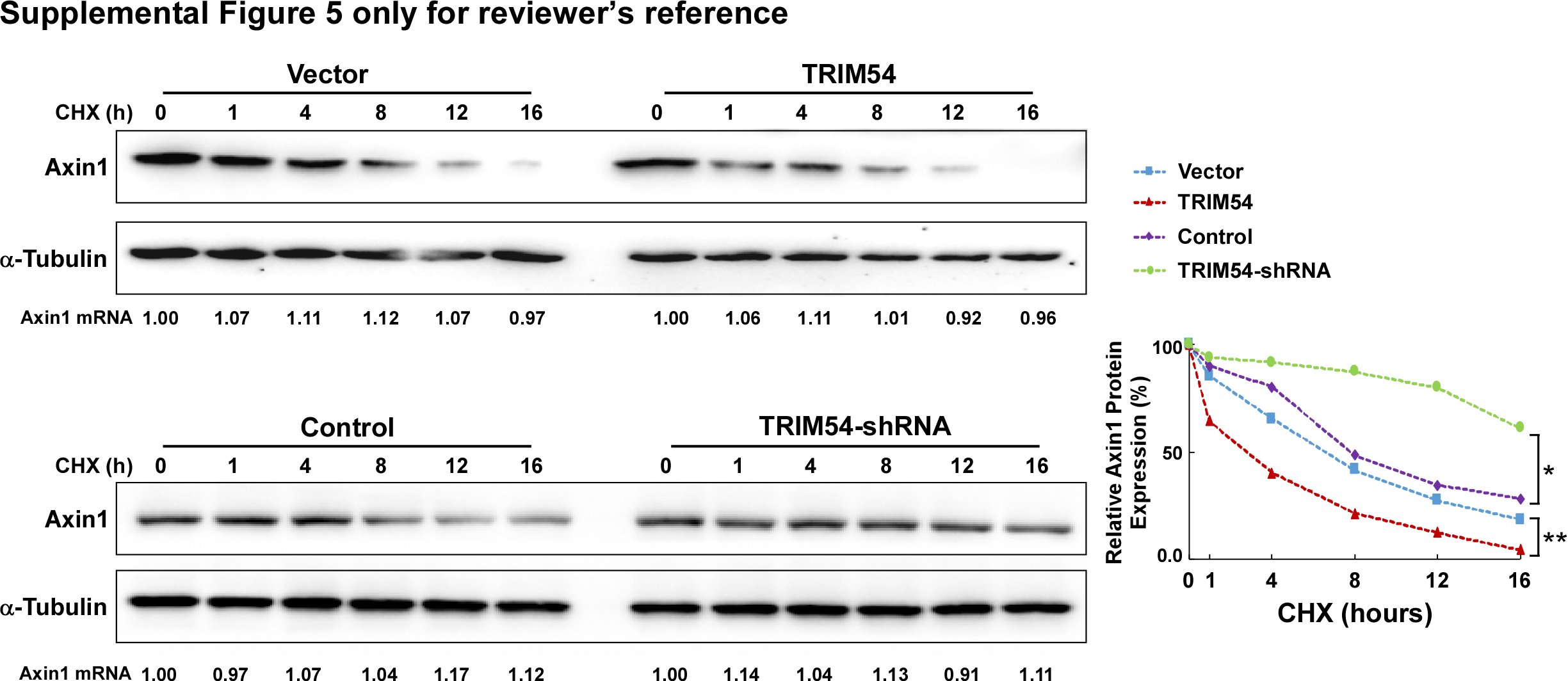

Supplement: Supplementary file 8 [file Image_6.tif]
